# Training Does Not Alter Muscle Ceramide and Diacylglycerol in Offsprings of Type 2 Diabetic Patients Despite Improved Insulin Sensitivity

**DOI:** 10.1155/2016/2372741

**Published:** 2016-09-29

**Authors:** Ditte Søgaard, Torben Østergård, Agnieszka U. Blachnio-Zabielska, Marcin Baranowski, Andreas Hansen Vigelsø, Jesper Løvind Andersen, Flemming Dela, Jørn Wulff Helge

**Affiliations:** ^1^Xlab, Centre of Healthy Aging, Department of Biomedical Sciences, University of Copenhagen, Copenhagen, Denmark; ^2^Department of Endocrinology and Diabetes M, Aarhus University Hospital, Aarhus Sygehus, Aarhus, Denmark; ^3^Department of Internal Medicine, Regional Hospital Viborg, Viborg, Denmark; ^4^Department of Physiology, Medical University of Bialystok, Bialystok, Poland; ^5^Institute of Sports Medicine Copenhagen, Bispebjerg Hospital, Copenhagen, Denmark

## Abstract

Ceramide and diacylglycerol (DAG) may be involved in the early phase of insulin resistance but data are inconsistent in man. We evaluated if an increase in insulin sensitivity after endurance training was accompanied by changes in these lipids in skeletal muscle. Nineteen first-degree type 2 diabetes Offsprings (Offsprings) (age: 33.1 ± 1.4 yrs; BMI: 26.4 ± 0.4 kg/m^2^) and sixteen matched Controls (age: 31.3 ± 1.5 yrs; BMI: 25.3 ± 0.7 kg/m^2^) performed 10 weeks of endurance training three times a week at 70% of VO_2_max on a bicycle ergometer. Before and after the intervention a hyperinsulinemic-euglycemic clamp and VO_2_max test were performed and muscle biopsies obtained. Insulin sensitivity was significantly lower in Offsprings compared to control subjects (*p* < 0.01) but improved in both groups after 10 weeks of endurance training (Off: 17 ± 6%; Con: 12 ± 9%, *p* < 0.01). The content of muscle ceramide, DAG, and their subspecies were similar between groups and did not change in response to the endurance training except for an overall reduction in C22:0-Cer (*p* < 0.05). Finally, the intervention induced an increase in AKT protein expression (Off: 27 ± 11%; Con: 20 ± 24%, *p* < 0.05). This study showed no relation between insulin sensitivity and ceramide or DAG content suggesting that ceramide and DAG are not major players in the early phase of insulin resistance in human muscle.

## 1. Introduction

The 21st century faces a major health challenge worldwide as the prevalence of insulin resistance (IR) and type 2 diabetes (T2D) has amplified extensively along with obesity [1, 2].

In obesity, lipid overload leads to increased levels of both plasma free fatty acids (FFA) and intramyocellular triglyceride (IMTG) and the latter has shown a negative correlation with insulin sensitivity [3, 4]. Similar to the obese condition, also healthy lean first-degree relatives of type 2 diabetic patients exhibit higher plasma fatty acid and IMTG concentration and a lower insulin sensitivity [4, 5]. There is at present no evidence for a causal link between IMTG and insulin sensitivity, but IMTG may facilitate an increased content of the bioactive lipids ceramide and diacylglycerol (DAG) in muscle, which have been suggested to mediate this link through an effect on insulin signaling. In short, ceramide is suspected to inhibit AKT phosphorylation by activation of protein kinase C*ζ* (PKC*ζ*) and protein phosphatase 2A (PP2A), while DAG is thought to activate protein kinase C*θ* (PKC*θ*), which inhibits insulin receptor substrate 1 [6–10].

Human studies have reported a higher muscle content of ceramide and lower insulin sensitivity in obese and type 2 diabetic patients compared to lean subjects [3, 11–13]. In line with this, a higher muscle ceramide content and lower insulin sensitivity was observed in Offsprings from type 2 diabetic patients compared to matched Controls [14]. However, in contrast, no difference in muscle ceramide content was observed in four groups that was comprised of age and lean body mass of matched trained, control and IGT subjects, and type 2 diabetic patients, thus representing markedly different insulin sensitivities [15]. Similarly, Helge et al. [16] found no difference in muscle ceramide content in trained versus untrained and sedentary subjects, respectively, while Chow et al. found a greater ceramide content in trained subjects than sedentary subjects [17], highlighting the discrepancy on ceramide data in human muscle. Also the data on DAG muscle content in man are somewhat inconsistent, where some studies reported higher muscle DAG content in obese and T2D compared to lean subjects [13, 14]. In contrast, Amati et al. [11] found lower muscle DAG content in obese compared to both lean sedentary subjects and athletes, whereas other studies showed no difference in muscle DAG content between lean, obese, and trained subjects [12, 13, 17]. Acute lipid infusion however increased total DAG content to a higher extent in lean compared to athletes [17]. In contrast, two studies from the Bergman group reported lower levels of DAG in muscle in endurance trained athletes compared with lean sedentary Controls, obese, and T2D subjects [18, 19].

**Figure 1:**
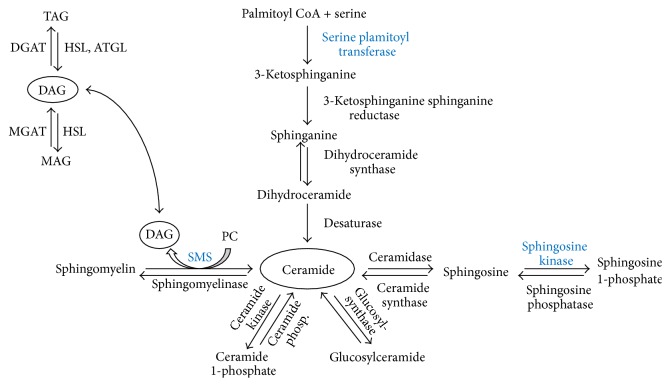
Ceramide and DAG metabolism in skeletal muscle. Ceramide is synthesized de novo from palmitoyl CoA and serine. Reversible reactions produce ceramide from sphingosine, glucosylceramide, ceramide 1-phosphate, and sphingomyelin, respectively. Conversion of ceramide to sphingomyelin also produces DAG. DAG is further synthesized from monoacylglycerol and from degradation of triacylglycerol. PC: phosphocholine; SMS: sphingomyelin synthase; MAG: monoacylglycerol; MGAT: monoacylglycerol acyltransferase; DGAT: diacylglycerol acyltransferase; HSL: hormone-sensitive lipase; ATGL: adipose triglyceride lipase. Proteins marked in bold blue are analyzed by western blotting.

Ceramide and DAG metabolism are controlled by several pathways (Figure 1), and the mechanisms regulating the content and composition of the subspecies in muscle are not fully elucidated. Ceramide can be synthesized de novo by serine palmitoyl transferase (SPT), which catalyzes the first and rate-limiting step [20, 21]. Ceramide is converted to sphingosine by ceramidase and further to sphingosine 1-phosphate (S1P) by sphingosine kinase (SphK) with both reactions being reversible [22]. In addition ceramide and phosphocholine can be reversibly converted to sphingomyelin and DAG by sphingomyelin synthase (SMS) [22]. In addition, DAG can be made from degradation of phospholipids or metabolized via the normal pathways where diacylglyceride acyltransferase (DGAT) converts DAG and FA to triacylglyceride (TAG) and conversely hormone-sensitive lipase (HSL) and adipose triglyceride lipase (ATGL) degrades TAG to DAG and FA and HSL further to monoacylglyceride and FA [23–25].

Training improves whole body glucose tolerance and insulin sensitivity in human skeletal muscle [26, 27], but the effect of training on muscle content of ceramide and DAG is not clear. Some studies found that training improved insulin sensitivity and decreased total muscle ceramide and DAG content and specific subspecies in sedentary overweight and obese subjects [28–30]. In contrast, we observed similar muscle ceramide content in trained insulin sensitive compared to untrained impaired glucose tolerant subjects [15]. Similarly, other groups have observed no changes in muscle ceramide or DAG content after 12 weeks of training in lean or obese women [31] and men [32]. Overall, there is conflicting and inconsistent data on both the basal content and the effect of training on muscle ceramide and DAG content in man and there is very limited data on the effects on the regulatory pathways in man.

Therefore, we aimed to study the influence of training and genetic susceptibility to T2D on muscle ceramide and DAG content and the regulatory pathways. Based on the inconsistent data available, we wanted to contribute with new knowledge in this area in order to elucidate the role of muscle ceramide and DAG in insulin resistance and type 2 diabetes.

## 2. Methods

### 2.1. Subjects

Nineteen first-degree Offsprings of type 2 diabetic patients (Offsprings) and sixteen Controls were included in the study. The inclusion was based on availability of muscle tissue before and after training from the full study group of twenty-nine and nineteen subjects, respectively, from a former study [33]. In brief, subjects were recruited at the outpatient clinic at Medical Department M at Aarhus University Hospital, Denmark, and through parents formerly diagnosed with T2D. Furthermore, some Offspring subjects and all the Controls were recruited through advertisements in local newspapers. None of the subjects were related. The subjects were matched based on the following criteria: Caucasian inheritance, 20–50 years old, body mass index (BMI) < 30 kg/m^2^, VO_2_max < 50 mL·min^−1^·kg^−1^, normal glucose tolerance, healthy and sedentary lifestyle, and not engaging in regular physical activity. The score of habitual activity level measured by Baecke questionnaire was similar between the groups [33]. The subjects were not allowed to take any form of prescribed medicine on regular basis. The Offspring and Control groups were matched by age, gender, and BMI and the Offsprings did not differ from the Controls metabolically in fasting plasma insulin, nonesterified fatty acids, or glucose. However, the Offsprings had a higher level of fasting plasma C-peptide [33].

The subjects gave written consent to participate in the study. The study was approved by the local committee of the county of Aarhus, Denmark, and the study complies with The Declaration of Helsinki. The data for the full study group describing the basic characteristics and the intervention has been published previously [33].

### 2.2. Experimental Design

The design and experimental procedures of the study have previously been described in detail and will here only be outlined in brief [33].

### 2.3. Training Protocol

The training intervention was performed over a 10-week period with three sessions a week. Each session consisted of 45 min. of nonsupervised aerobic exercise where the subjects were instructed to aim for a heart rate corresponding to 70% of their VO_2_max. The exercise was performed on a bicycle ergometer at a local fitness center or on a provided bicycle ergometer at home. After five weeks of training, the VO_2_max was measured and the exercise work load adjusted. The adjustments were similar between groups and negligible.

### 2.4. Intervention

Prior to the 10-week training intervention, a maximal exhaustive incremental test on a bicycle ergometer and a submaximal test admodum, Astrand et al. [34], were performed. The highest test result was selected as the baseline VO_2_max. The exercise test protocol was performed both before and after the intervention. Insulin sensitivity was measured by a hyperinsulinemic-euglycemic clamp and muscle biopsies obtained at initiation and again 3-4 days after the last exercise session. In addition, a standard 2-hour oral glucose tolerance test (OGTT) was carried out prior to the intervention and repeated 4-5 days before the last exercise bout.

### 2.5. Hyperinsulinemic-Euglycemic Clamp

After an overnight fast, the subjects rested in bed for 150 min. After 120 min., muscle biopsies were obtained. Insulin was infused starting at 150 min. at a rate of 1.0 mU·kg^−1^·min^−1^ and plasma glucose was kept at 5 mM by glucose infusion (200 mg/L). Steady state was achieved after 270–300 min. (120–150 min. into the clamp) and the average glucose infusion rate was taken as the insulin stimulated glucose uptake (ISGU). Energy expenditure and glucose and lipid oxidation rates were measured between 120–150 and 270–300 min., respectively, using an open-circuit ventilation hood.

### 2.6. Muscle Biopsies

Muscle biopsies were taken in vastus lateralis of the quadriceps muscle by a Bergstrom needle (5 mm) after injection of local anaesthesia (10% lidocaine) in the skin and percutaneous region. The biopsy was divided and immediately put into −80°C liquid nitrogen or mounted with Tissue-Tek (Sakura Finetek, Zoeterwoude, Netherlands) and frozen in isopentane cooled in liquid nitrogen. The samples were subsequently stored at −80°C for analysis. Prior to analyses, visible blood and fat were removed from the biopsies.

### 2.7. Bioactive Lipid Analysis

Ceramides and diacylglycerols were measured according to the methods described by Blachnio-Zabielska et al. [35, 36]. Briefly, lipids were extracted from ~20 mg of tissue by the use of the extraction mixture composed of isopropanol : water : ethyl acetate (35 : 5 : 60; v : v : v). Quantitative measurement of ceramides and diacylglycerols was made using an Agilent 6460 triple quadrupole mass spectrometer. Both ceramides and diacylglycerols were analyzed using positive ion electrospray ionization (ESI) source with multiple reaction monitoring (MRM). The chromatographic separation was performed using an Agilent 1290 Infinity Ultra Performance Liquid Chromatography (UPLC). The analytical column was a reverse-phase Zorbax SB-C8, 2.1 × 150 mm, 1.8 *μ*m (Agilent, Santa Clara, CA). Chromatographic separation was conducted in binary gradient using 2 mM ammonium formate, 0.15% formic acid in methanol as Solvent A, and 1.5 mM ammonium formate; 0.1% formic acid in water as Solvent B at the flow rate of 0.4 mL/min. C17:0-ceramide and 1,3-dipentadecanoyl-rac-glycerol (Avanti Polar Lipids, Alabaster, AL) were used as internal standards for ceramides and diacylglycerols, respectively. The HPLC grade methanol and water as well as formic acid and ammonium formate were obtained from Sigma-Aldrich (St. Louis, MO).

### 2.8. Western Blot

To measure the content of proteins involved in sphingolipid metabolism or insulin signaling western blotting was performed as described previously [37] with modifications. In brief, muscle biopsies were freeze-dried for 48 hours at −40°C at <0.5 mBar followed by 1 hour of equilibration to room temperature (RT) at maintained pressure. ~20 mg tissue (wet weight) was dissected and homogenized in 400 *μ*L cold Radio-Immunoprecipitation Assay buffer added protease and phosphate inhibitors. Protein concentration was measured in triplicate using a bicinchoninic acid (BCA) assay. 10 *μ*g of protein per 13 *μ*L homogenate was diluted in Laemmli buffer and MilliQ water. The tubes were heated and the samples and a calibrator (5 *μ*g), a molecular weight marker (Magicmark, XP western std. (2 *μ*L), and High Range Rainbow molecular weight marker (2 *μ*L)) were loaded and separated on 26 wells 12% Criterion TGX Stain-Free polyacrylamide SDS gels at 100 V. The gels were activated and a 1 sec. image was taken with LAS 4000 image analyzer (GE Healthcare, Little Chalfont, UK). The gels were transferred to an ethanol activated polyvinylidene fluoride (PVDF) membrane (0.2 *μ*m pores, Bio-Rad, Copenhagen, Denmark) using semidry blotting at 25 V in 7 min. A 1 sec. image was taken with UV light of the membranes with the proteins transferred.

The membranes were blocked in 5% skimmed milk or 5% bovine serum albumin (BSA) diluted in Tris-buffered saline (TBS) for 1 or 1.5 hours at RT. The membranes were incubated overnight with primary antibody: antiserine palmitoyl transferase (SPT) 1 : 2000 (ab23696, Abcam, Cambridge, UK), antisphingomyelin synthase 2 (SMS2) 1 : 2000 (ab103060, Abcam), antisphingosine kinase 1 (SphK1) 1 : 1000 (ab37980, Abcam), and anti-AKTpan 1 : 1000 (number 4691, (C67E7), Cell Signaling, Massachusetts, USA) all in 5% skimmed milk, and anti-p-AKT (ser473) 1 : 1000 (number 4060, (D9E), Cell Signaling), antiprotein kinase C*θ* (PKC*θ*) 1 : 2000 (ab110728, Abcam), and anti-p-PKC*θ* (ser676) 1 : 2000 (ab131479, Abcam) all in 5% BSA. The secondary antibody used was polyclonal goat anti-rabbit horseradish peroxidase conjugated (number 7074S, Cell Signaling) 1 : 2000 diluted in 5% skimmed milk or 5% BSA in TBS in line with the primary antibody. The membranes were washed 2 × 5 min. with TBS added 0.05% Tween 20 followed by 1 × 5 min. with TBS after incubation with primary and secondary antibody, respectively. The blots were incubated for 1 min. with ECL detection reagents (Amersham western blotting detection reagents, GE Healthcare, UK) and the proteins visualized. The UV images of the membranes and the images of the proteins of interest were quantified using ImageQuant TL software version 7.0 (GE Healthcare). The intensity of each band of interest was normalized to total protein measured by Stain-Free fluorescence (UV picture after transfer) as described previously [38]. To compare the samples loaded on different gels, all samples were quantified relative to the calibrator (pool of all samples) which was loaded on all gels in 2-3 lanes.

### 2.9. Fiber Type Distribution

Muscle fiber type distribution was performed and analyzed using myofibrillar ATPase histochemistry as previously described [33].

### 2.10. Statistical Analyses

All data are presented as means ± SEM. Comparison of the Control and Offspring groups and the effect of the intervention were analyzed by a two-way analysis of variance (ANOVA) with repeated measurements and Holm-Sidak post hoc test. Correlations were carried out using Pearson Correlation Coefficient. All analyses were performed using Sigma Plot 12.5.

## 3. Results

The compliance of the subjects to the exercise program was similar between the groups according to number of training sessions performed, as previously described [33]. Maximal oxygen uptake, body weight, insulin sensitivity, and insulin basal during the clamp were reported in a prior paper [33], but they are also included here with the values of the subset of persons that are included in this paper. Ten weeks of training induced a decrease (*p* = 0.003) in body weight of 1.1 ± 0.3 kg (Table 1), which was independent of group and gender. Prior to the experiment, VO_2_max was not different between the Control and Offspring groups, and, after the training, VO_2_max was increased (*p* < 0.001) similarly in the groups by 16 ± 2% (Table 1). The insulin sensitivity, measured as *M*-value, was higher (*p* = 0.008) in the Control compared to the Offspring group and after 10 weeks of exercise training it was increased (*p* = 0.008) by 17.0 ± 5.9% and 11.8 ± 9.2% in Offspring and Control, respectively (Table 1). Insulin sensitivity did not differ (*p* = 0.484) between males and females (data not shown).

As described previously [33] a positive correlation was found between insulin sensitivity and VO_2_max at baseline and after the exercise training intervention in the Control group.

### 3.1. Muscle Protein

**Figure 2:**
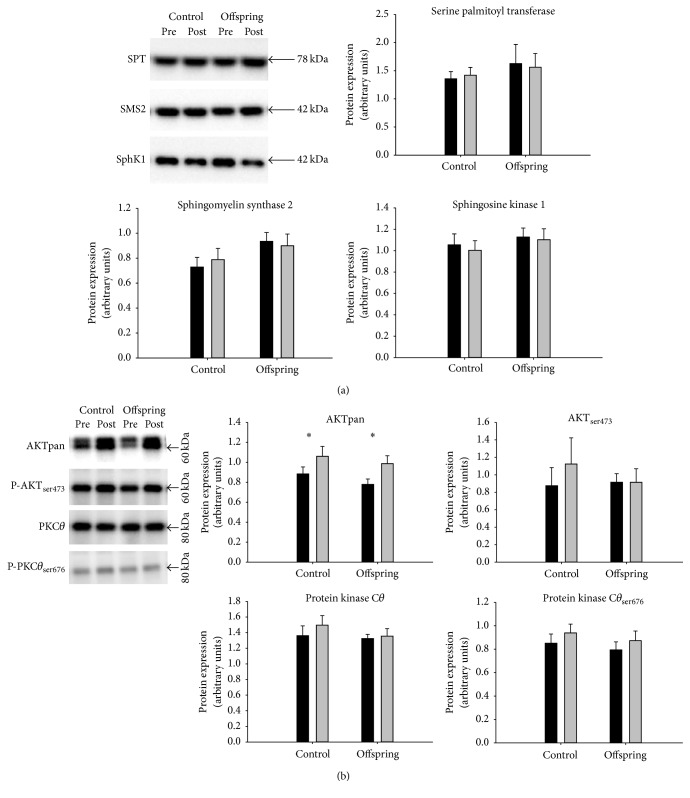
Protein expression including representative blots in Offsprings of type 2 diabetic patients and matched Controls before and after 10 weeks of training intervention. Proteins presented are involved in (a) ceramide and DAG metabolism and (b) insulin signaling. Pre: black bars; Post: grey bars. *∗*: main effect of the training intervention in Offspring and Control subjects (*p* = 0.03, *n* = 21).

**Figure 3:**
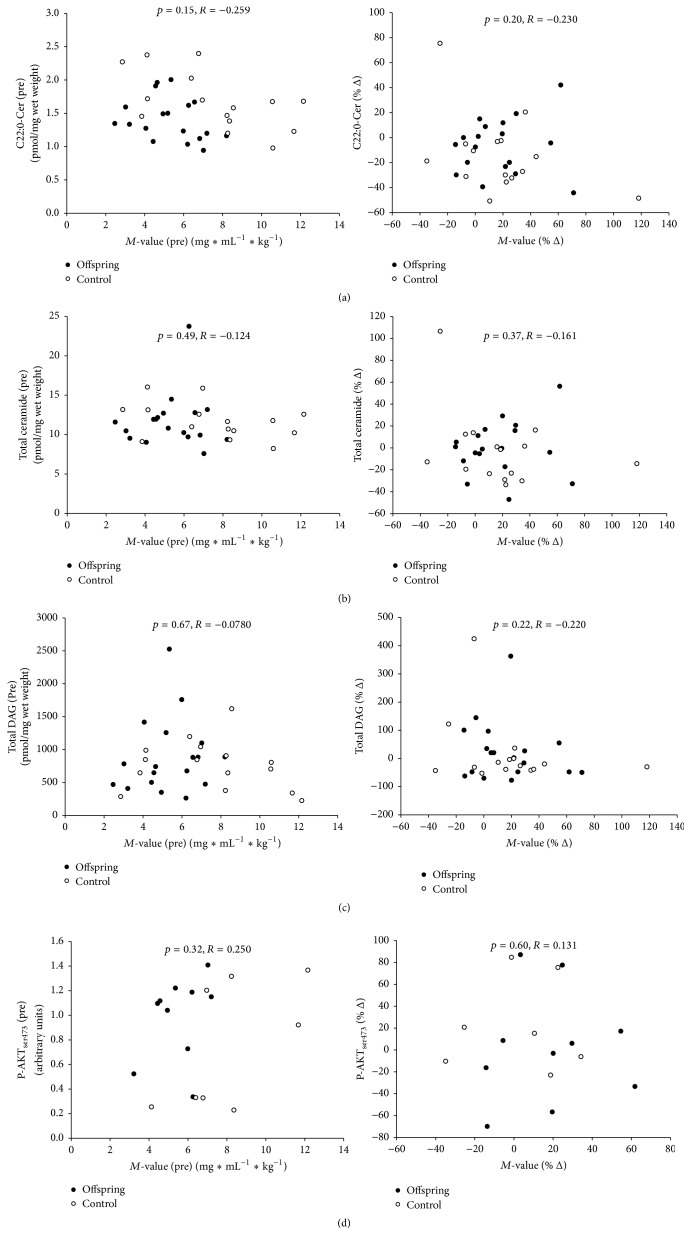
Muscle content of (a) C22:0-Cer, (b) total ceramide, (c) total DAG, and (d) P-AKT_ser473_ protein expression as function of insulin sensitivity at basal and the percent change after 10 weeks of endurance training in type 2 diabetic Offsprings (●) and matched Controls (○).

The muscle protein content of basal SPT, SMS2, SphK1, PKC*θ*, P-PKC*θ*_ser676_ and P-AKT_ser473_ did not differ between the groups and was not affected by the intervention (Figure 2). Basal AKT protein expression did not differ between the groups but increased (*p* = 0.03) in both groups after the intervention (Figure 2(b)). AKT_ser473_ did not correlate with insulin sensitivity at basal or as % change after the intervention (Figure 3(d)). The individual percent change in SMS2 protein expression after the training intervention was positively correlated (*p* = 0.006, *R* = 0.582, *n* = 21) with the change in SphK1.

### 3.2. Muscle Bioactive Lipids

The total muscle ceramide content and the content of ceramide subspecies were not different between the Offspring and Control group or between genders (Table 2). There was no difference in C16:0-Cer content between the groups and no main effect on the change in C16:0-Cer content, but a borderline significant training × group interaction (*p* = 0.082) was found. Due to lack of a significant interaction we could not further test for differences (Table 2), but a numerical reduction of 33% was observed in the Control group and 3.2% in the Offsprings. The total ceramide content was not changed (*p* = 0.27) in response to 10 weeks of training in either of the groups (Table 2). However, C22:0-Cer was significantly and similarly reduced (*p* = 0.02) in the two groups after the intervention (Table 2). The content of total ceramide (*p* = 0.49, *R* = −0.124) or C22:0-Cer (*p* = 0.15, *R* = −0.259) did not correlate with insulin sensitivity before (Figures 3(a) and 3(b)) or after (figure not shown) the endurance intervention. The calculated individual % differences in total ceramide (*p* = 0.37, *R* = −0.161) and C22:0-Cer (*p* = 0.20, *R* = −0.230) content across the training period were not correlated with the percent change in insulin sensitivity (Figures 3(a) and 3(b)). The degree of ceramide saturation did not change in response to the training and did not correlate with insulin sensitivity. No correlations were found either between the % change in content of any of the proteins measured or total ceramide content.

The total DAG content and the content of the individual DAG subspecies in both Offspring and Control subjects were not significantly influenced by the training intervention (Table 3). Moreover, there was no difference between the two groups in muscle content of total DAG or individual subspecies (Table 3) or between genders. Furthermore, the total content of DAG measured in muscle was not correlated with insulin sensitivity at baseline (*p* = 0.67, *R* = −0.078), after the intervention (figure not shown), or across the intervention (*p* = 0.22, *R* = −0.220), calculated as the individual % differences (Figure 3(c)). Similarly, the DAG subspecies or the degree of saturation of DAG (data not shown) did not correlate with insulin sensitivity at any time point. The % change in total DAG content was not correlated with the % change in any of the proteins measured.

### 3.3. Fiber Type

Fiber type data were reported in a prior paper [33], but they are also included here for the subset of persons that are included. The muscle fiber type distribution did not differ between the groups or in response to the exercise training intervention (Table 1).

## 4. Discussion

A major finding of this study was that the key proteins in the regulatory pathways of ceramide and diacylglycerol metabolism were not different between Offspring of type 2 diabetic patients and matched Control subjects or influenced by training. In addition, we observed that ceramide and DAG muscle content and subspecies, except for ceramide 22:0 subspecies, were not affected by 10 weeks of training in Offspring of type 2 diabetic patients and matched Control subjects, though a clear training induced increase in insulin sensitivity and maximal oxygen uptake as well as increased citrate synthase and cytochrome oxidase activity, as previously described [33]. Finally, there were no differences between groups or sex in ceramide and DAG content and their subspecies.

The observation of no effect of training on total muscle ceramide and DAG content in the present study is in line with previous findings, showing no difference in ceramide and DAG content between lean and obese women [31] or men [32] after 12 weeks of endurance training or ceramide content after 15 weeks of life style intervention [39], though the training and lifestyle induced improvement in insulin sensitivity in these three studies. Yet, Dubé et al. found a decrease in ceramide and DAG content in older overweight to obese subjects in response to 16 weeks of endurance training [28, 29]. In addition, a recent study by Coen et al. found a lower ceramide content after gastric bypass induced weight loss and exercise compared to just gastric bypass induced weight loss [40]. Evidently, the available data on the effect of training on muscle bioactive lipid content are very inconsistent and an explanation for this is not readily apparent. We have previously demonstrated that ceramide content was higher in type I than type II muscle fibers in man [41, 42] and therefore fiber type distribution, which often varies between lean and obese [28, 43] and which in vastus lateralis demonstrate rather large heterogeneity [44], may explain some of this inconsistency. The total content of ceramide and DAG does not decrease significantly in response to endurance training; however, it could also be speculated that small nonsignificant changes may be biologically relevant. The lack of training induced changes could be explained by a low power, but previous studies have reported changes with much fewer subjects included [28, 29]. Furthermore, it should be kept in mind that insulin sensitivity may be affected by mitochondrial dysfunction, impaired beta oxidation, and ROS emission as well as inflammation and this could potentially mask a possible relationship between ceramide and DAG and insulin sensitivity. It could further be emphasized that the total content of ceramide and DAG in muscle is not important in insulin resistance and that we should turn our attention to specific subspecies or specific localization. In the present study, all the ceramide and DAG subspecies were unchanged except for C22:0-Cer, where we found a significant reduction, yet the change was not correlated with the improvement in insulin sensitivity measured by the euglycemic hyperinsulinemic glucose clamp technique. Prior studies [29, 40] have not reported C22:0-Cer as a subspecies suspected to affect insulin sensitivity so the observed reduction in C22:0-Cer content may not be of great importance in this context. The C16:0-Cer subspecies has been associated with reduced insulin sensitivity in some studies [12, 29], while others do not find a link [11]. We did not detect significant changes in C16:0-Cer after endurance training, but a borderline significant (*p* = 0.082) interaction between the groups indicates that the 33% numerical reduction in C16:0-Cer in the control group could be significant and may be of biological relevance. Coen and colleagues recently reported changes in specific ceramide species when gastric bypass induced weight loss was combined with exercise [40] and the same group also observed differences in ceramide and DAG subspecies in a cross-sectional study of trained versus untrained [11] and with diet induced weight loss and exercise [29]. Reductions in specific ceramide and DAG subspecies have furthermore been found after 8 weeks of endurance training intervention in obese subjects including C16:0-Cer [30]. The inconsistency in the exercise induced effect on the content of total ceramide and subspecies is not readily explainable, but it is indeed possible that specific localization of subspecies within the cell may exert a modulatory effect on insulin signaling as recently suggested for DAG species [18, 45]. A study by Boon et al. reported that induction of insulin resistance in mice by ceramide infusion only increased ceramide in the plasma membrane of the myocyte supporting this hypothesis [46]. However, the actual mechanisms for this in man remain largely unknown and further work is needed to confirm and outline this.

We found a small but significant reduction in body weight which could potentially have affected insulin sensitivity and metabolism. However, it is a relatively small change and, taking day to day variation into account, this probably does not play an important role in insulin sensitivity.

As published in the initial study [33], Offsprings had lower insulin sensitivity than Controls consistent with findings in other studies [14, 47, 48]. If ceramide and/or DAG in muscle plays a role in the early phase of insulin resistance, we would have expected to find higher levels in the Offspring group, and this was not the case. In contrast, Straczkowski et al. reported that ceramide content was increased in type 2 diabetes offspring subjects [14]. These contradicting results may in part be explained by the offspring subjects displaying different metabolic conditions and developmental stages of insulin resistance. Furthermore, it should be noted that not all type 2 diabetes Offsprings develop insulin resistance [4, 49].

The similar protein expression of the three key enzymes in ceramide and DAG metabolism agrees well with the lack of differences in ceramide and DAG total content and their subspecies between the groups and in response to the training. However, the activity of these enzymes may not only depend on the expression level but could also be regulated by posttranslational modification or association with regulatory proteins. The content of SMS2 and SphK1 was not significantly affected by training, but there was a strong positive correlation between the percent change in expression of SMS2 and SphK1, suggesting that the protein expression of the pathway is linked.

We found no differences in basal AKT protein expression and p-AKT_ser473_ between Offsprings and Controls which is consistent with studies demonstrating no difference in expression of AKT and p-AKT_ser473_ between lean, obese insulin resistant, and nondiabetic insulin resistant Offsprings of type 2 diabetic patients and type 2 diabetic patients [3, 50, 51]. In contrast, one study observed a lower basal AKT protein content in diabetic patients compared to Controls [52]. Studies have reported an increase in basal AKT protein expression in response to aerobic training of three to ten weeks in duration in healthy males, control, obese, and T2D subjects [52, 53]. These findings agree well with our results and are also in concordance with the observed improvement in insulin sensitivity. Protein expression of p-AKT_ser473_ in muscle has shown to increase in response to insulin stimulation in muscle in healthy, obese, and type 2 diabetic subjects, but whether the insulin stimulated p-AKT_ser473_ content differ between subjects displaying different metabolic conditions is not clear [51–54]. The basal content of p-AKT_ser473_ has been reported not to change in response to exercise training, while it is a bit more controversial in the insulin stimulated state showing an increase or no change [52–54].

In a recent study, Szendroedi et al. reported higher PKC*θ* activity and total and cytosolic DAG content in obese and T2D subjects compared to Controls, while membrane DAG was only increased in T2D [55]. Nevertheless, we found no differences in PKC*θ* or p-PKC*θ*_ser676_ protein expression between Controls and Offsprings and no training induced changes either. In agreement with this, Rose et al. showed no changes in cytosolic or membrane PKC*θ* protein content in muscle in young untrained subjects after one bout of exercise of 40 min. at 76 ± 1% of VO_2_peak [56]. If indeed DAG inhibits insulin signaling by activation of PKC*θ* as suggested [7, 8, 55], these findings are well in line with the lack of effects on DAG content in the present study. Increased plasma fatty acid content is usually linked to reduced insulin sensitivity and increased ceramide and DAG synthesis. Montell et al. found that human cultured cells treated with saturated FA induced changes in basal glucose uptake and insulin response that were related to DAG accumulation and PKC activation [7]. In line with this, lipid infusion was reported to induce an increase in DAG content and PKC*θ* activation in muscle in healthy young males and PKC*θ* activation was positively correlated with both plasma FA concentration and muscle DAG content and negatively correlated with insulin sensitivity [55]. However, in the present study, we found no differences in plasma FA concentration, DAG content, or PKC*θ* protein expression between Controls and Offsprings despite reduced insulin sensitivity, and this highlights the difficulty in comparing results from studies with acute exposure to lipids with chronic physiological conditions in human.

The general lack of change in content of proteins measured involved in insulin signaling except for AKT may indicate that changes in insulin signaling are not involved in the training induced improvement in insulin sensitivity. However, changes in insulin signaling may not exclusively be explained by the content of the proteins involved but also the activity of the proteins measured as the membrane to cytosol ratio.

A possible limitation to this study is that the biopsies originate from a study performed approximately 10 years ago, so the biopsies have been stored in −80°C freezer for a long period. However, four recently obtained biopsies from matched patients were applied as control biopsies and thus analyzed by lipidomics along with the biopsies from this study. We measured no difference in the values of ceramide and DAG content from the biopsies of this study compared to the recently obtained control biopsies. This confirms that the lipids are highly preserved in the older biopsies and that the values measured are reliable. Furthermore, our data indicates that the protein content also remains stable during the long term freezing period, since we observed similar band intensity of the specific proteins measured by western blot in the biopsies from the present study compared with biopsies from a recently completed study.

## 5. Conclusion

The total muscle content of ceramide and DAG and their measured subspecies are not different in first-degree Offsprings of type 2 diabetic patients compared to matched Controls. Ten weeks of exercise training improves VO_2_max, insulin sensitivity, and AKT protein expression in both Offsprings and Controls but does not induce any changes in ceramide or DAG content in line with our hypothesis. We found no correlation between insulin sensitivity and ceramide or DAG content, suggesting that ceramide and DAG are not major players in the early phase of insulin resistance in human skeletal muscle.

## Figures and Tables

**Table 1:** Subject characteristics, maximal oxygen uptake, and insulin sensitivity before and after 10 weeks of endurance training in T2D offspring and matched controls.

Characteristics	Control		Offspring	
	(*n* = 16)		(*n* = 19)	
	Pre	Post	Pre	Post
Gender (M/F)	10/6		12/7	
Age (yrs)	31.3 ± 1.5		33.1 ± 1.4	
Weight (kg)	80.6 ± 3.7	79.2 ± 3.5^*∗∗*^	83.7 ± 1.9	82.8 ± 1.8^*∗∗*^
BMI (kg/m^2^)	25.3 ± 0.7	24.9 ± 0.7	26.4 ± 0.4	26.1 ± 0.4
VO_2_max (mL/min)	3301 ± 198	3768 ± 266^*∗*^	3222 ± 179	3734 ± 208^*∗*^
VO_2_max (mL/min/kg)	41.1 ± 1.6	47.4 ± 2.2^*∗*^	38.3 ± 1.6	44.8 ± 2.0^*∗*^
*M*-value (mg/min/kg)	7.58 ± 0.76	8.47 ± 0.73^*∗∗*†^	5.35 ± 0.37	6.27 ± 0.52^*∗∗*^
Fiber type I%	46.3 ± 4.0	45.3 ± 3.4	46.3 ± 4.2	44.8 ± 4.1
Fiber type IIA%	37.1 ± 3.8	38.4 ± 2.5	34.7 ± 2.4	40.5 ± 2.7
Fiber type IIX%	16.6 ± 2.1	16.3 ± 2.7	18.9 ± 3.4	14.7 ± 2.4

The present data are based on subsample of previously published data [33]. Data are mean ± SEM. ^*∗*^Pre versus post main effect (*p* < 0.001);

^*∗∗*^Pre versus post main effect (*p* < 0.01); ^†^Con. versus Off. (*p* = 0.008).

**Table 2:** Muscle ceramide subspecies and total content before and after 10 weeks of endurance training in T2D offsprings and matched controls.

Ceramide	Control (*n* = 16)			Offspring (*n* = 19)			*p* value	
Subspecies	Pre	Post	% Δ	Pre	Post	% Δ	Training main effect	Training × group interaction
C14:0	0.0052 ± 0.0010	0.0046 ± 0.0007	−11.5	0.0042 ± 0.0009	0.0057 ± 0.0011	35.1	0.335	0.268
C16:0	0.41 ± 0.06	0.27 ± 0.04	−33.2	0.38 ± 0.09	0.37 ± 0.05	−3.22	0.251	0.082
C18:0	5.67 ± 0.36	5.55 ± 0.68	−2.08	6.06 ± 0.58	6.04 ± 0.27	−0.28	0.830	0.426
C18:1	0.12 ± 0.02	0.11 ± 0.02	−12.4	0.11 ± 0.01	0.11 ± 0.01	5.80	0.670	0.292
C20:0	0.046 ± 0.014	0.031 ± 0.004	−32.0	0.029 ± 0.003	0.029 ± 0.003	−0.01	0.384	0.286
C22:0	1.63 ± 0.11	1.39 ± 0.14^*∗*^	−14.8	1.38 ± 0.08	1.29 ± 0.08^*∗*^	−6.93	0.017^*∗*^	0.294
C24:0	0.0041 ± 0.0008	0.0036 ± 0.0016	−11.5	0.0033 ± 0.0007	0.0044 ± 0.0008	35.1	0.335	0.268
C24:1	3.81 ± 0.21	3.84 ± 0.29	0.94	3.58 ± 0.18	3.27 ± 0.24	−8.71	0.251	0.323
Mean % Δ			−14.6 ± 4.4			7.11 ± 6.3		
Total cer.	11.7 ± 0.6	11.2 ± 1.1	−4.27	11.6 ± 0.8	11.1 ± 0.5	−4.31	0.272	0.579

Data are means ± SEM. All data are given as pmol/mg wet weight. The statistics are based on log⁡10 due to failed normality testing. ^*∗*^Pre versus post main effect, (*p* = 0.017). The % delta calculated as the relative change from baseline.

**Table 3:** Muscle diacylglycerol subspecies before and after 10 weeks of endurance training in T2D offspring and matched controls.

DAG	Control (*n* = 16)			Offspring (*n* = 19)			*p* value	
Subspecies	Pre	Post	% Δ	Pre	Post	% Δ	Training main effect	Training × group interaction
16:0/16:0	152 ± 25	115 ± 14	−24.1	147 ± 21	140 ± 19	−5.01	0.291	0.480
16:0/18:0	213 ± 28	189 ± 26	−11.6	240 ± 42	239 ± 38	−0.48	0.695	0.720
16:0/18:1	211 ± 27	194 ± 24	−7.95	268 ± 40	260 ± 37	−3.00	0.666	0.940
16:0/18:2	33.9 ± 4.5	28.8 ± 3.5	−15.2	38.8 ± 6.1	35.3 ± 3.9	−9.05	0.338	0.855
18:0/18:1	24.4 ± 3.3	24.3 ± 2.9	−0.22	30.3 ± 3.6	29.6 ± 4.3	−2.37	0.911	0.923
18:1/18:1	74.4 ± 10.8	69.7 ± 9.7	−6.32	89.9 ± 13.9	87.1 ± 12.9	−3.16	0.729	0.932
18:1/18:2	50.1 ± 7.6	47.5 ± 6.6	−5.29	57.6 ± 8.6	51.1 ± 6.1	−11.3	0.453	0.751
Mean % Δ			−10.1 ± 2.9			−4.9 ± 1.6		
Total DAG	759 ± 90	668 ± 80	−12.0	872 ± 127	842 ± 114	−3.44	0.495	0.839

Data are means ± SEM. All data are given as pmol/mg wet weight. The statistics for 16:0/18:1 and total DAG are based on log⁡10 due to failed normality testing. The % delta calculated as the relative change from baseline.

## Abbreviations

ATGL: Adipose triglyceride lipase
DAG: Diacylglycerol
DGAT: Diacylglyceride acyltransferase
ESI: Electrospray ionization
FFA: Free fatty acids
HSL: Hormone sensitive lipase
IMTG: Intramyocellular triglyceride
IR: Insulin resistance
ISGU: Insulin stimulated glucose uptake
PKC: Protein kinase C
PP2A: Protein phosphatase 2A
S1P: Sphingosine 1-phosphate
SMS: Sphingomyelin synthase
SphK: Sphingosine kinase
SPT: Serine palmitoyl transferase
T2D: Type 2 diabetes
TAG: Triacylglyceride
UPLC: Ultra Performance Liquid Chromatography.
